# Subclassification-Specific Tumor Immune Microenvironment in Intrahepatic Cholangiocarcinoma: Implications for Appropriate Pharmacotherapy

**DOI:** 10.3390/cancers17132082

**Published:** 2025-06-21

**Authors:** Masahiko Kinoshita, Yasunori Sato, Shoji Kubo, Hiroji Shinkawa, Kenjiro Kimura, Kohei Nishio, Ryota Tanaka, Shigeaki Kurihara, Takeaki Ishizawa

**Affiliations:** 1 Department of Hepato-Biliary-Pancreatic Surgery, Osaka Metropolitan University Graduate School of Medicine, Osaka 545-8585, Japan; kubosho65@yahoo.co.jp (S.K.); hirojishinkawa9876@gmail.com (H.S.); kenjiro@omu.ac.jp (K.K.); m1155123@omu.ac.jp (K.N.); taanaakaa3364@gmail.com (R.T.); s-kurihara0731@hotmail.com (S.K.); take1438@gmail.com (T.I.); 2 Department of Human Pathology, Kanazawa University Graduate School of Medical Sciences, Kanazawa 920-8640, Japan; 3 Health Education Course, Department of Education, Faculty of Education, Shitennoji University, Osaka 583-8501, Japan

**Keywords:** cholangiocarcinoma, prognosis, CD8-Positive T-Lymphocytes, dendritic cells, programmed cell death 1 receptor, antigen presentation, S100 protein

## Abstract

**Background/Objectives**: Intrahepatic cholangiocarcinoma (iCCA) is subclassified into small- and large-duct types. Small-duct-type iCCAs are associated with a better prognosis, and each subclassification requires different surgical strategies. The efficacy of chemotherapy, including immune checkpoint inhibitors, may vary between subclassifications. However, there are no reports on tumor immune microenvironment (TIME) analyses based on iCCA subclassifications. This study investigated subclassification-specific TIMEs in iCCAs for the purpose of establishing appropriate pharmacotherapy. **Methods**: A total of 131 resected iCCA cases were analyzed, comprising 73 tumors classified as small-duct-type and 58 as large-duct-type based on pathological evaluation. Immunohistochemical analyses targeting CD8, PD-1, PD-L1, CTLA-4, and S100 protein (a dendritic cell [DC] marker) were performed to investigate the immune-cell status in each subclassification. **Results**: Large-duct-type iCCA had a significantly higher CD8 expression in tumor-infiltrating cells than small-duct-type ICC. However, the expression of other molecules did not significantly differ between the two tumor types. The proportion of tumors with a high level of S100 protein expression (DC-high group) in tumor-infiltrating cells was significantly higher in small-duct-type ICCs than in large-duct-type iCCAs (30% vs. 1.7%). In small-duct-type iCCAs, the expression levels of CD8, PD-1, PD-L1, and CTLA-4 were significantly higher in the DC-high group than in the DC-low group. **Conclusions**: We revealed subclassification-specific TIMEs in iCCAs. A subset of small-duct-type iCCAs exhibited strong DC infiltration. In these patients, the tumors may establish an immunosuppressive TIME to evade antitumor immunity triggered by DC-mediated antigen presentation. These findings may contribute to the development of tailored pharmacotherapy for each iCCA subclassification.

## Simple Summary

Intrahepatic cholangiocarcinoma (iCCA) is subclassified into small- and large-duct types, and several clinicopathological differences between subclassifications have been reported. However, there are no reports on tumor immune microenvironment (TIME) analyses based on iCCA subclassifications. This study investigated subclassification-specific TIMEs in iCCAs for the purpose of establishing appropriate pharmacotherapy. The results indicate that there is a group of strongly infiltrated DCs in small duct-type iCCA, and in such patients, iCCAs may establish an immunosuppressive TIME to escape from antitumoral immunity induced by the antigen presentation of DCs. The diversity in background factors and the anatomical origins of iCCAs not only contribute to the formation of distinct subclassifications with different clinicopathological characteristics, but also influence the composition of the TIME. This result may contribute to the establishment of appropriate pharmacotherapy for each subclassification.

## 1. Introduction

Intrahepatic cholangiocarcinoma (iCCA) is a malignant epithelial tumor arising from the intrahepatic bile ducts, and represents the second most common primary liver malignancy. In the 5th Edition of the World Health Organization (WHO) Classification of Tumors, published in 2019, iCCAs were subclassified into small- and large-duct types according to their anatomical origin. Large-duct-type iCCAs typically arise near the hepatic hilum and are characterized grossly by periductal infiltration or a combination of periductal infiltration and mass-forming patterns [1,2,3,4]. Further, their risk factors are primary sclerosing cholangitis, hepatolithiasis, liver flukes, and exposure to chlorinated organic solvents, such as 1.2-dichloropropane [1,5]. Large-duct-type iCCAs are frequently observed in the perihilar region, and they metastasize to the lymph node [6,7]. In contrast, small-duct-type iCCAs are primarily located in the periphery of the liver parenchyma. Grossly, this tumor type resembles the mass-forming type. Similarly to those for hepatocellular carcinoma, the risk factors for this iCCA type include nonbiliary cirrhosis and chronic viral hepatitis. These clinicopathological differences suggest that each iCCA subclassification requires a different surgical strategy [8].

In recent years, the efficacy of immune checkpoint inhibitors (ICIs) has been widely recognized. ICIs have become an important treatment option for various cancer types [9,10,11]. The TOPAZ-1 and KEYNOTE-966 trials showed that the addition of ICIs to gemcitabine and cisplatin significantly improved patient outcomes. Hence, this therapeutic regimen is a promising pharmacotherapeutic approach for unresectable, advanced-stage, or recurrent ICC [12,13]. Biomarkers such as tumor-infiltrating lymphocytes in the tumor immune microenvironment (TIME), programmed death-1 ligand (PD-L1) expression, KRAS alteration, and TGFBR2 may predict the efficacy of pharmacotherapy including ICIs for biliary tract cancer [14]. Therefore, responses to ICIs differ across cases. Identifying predictive factors with greater clinical utility for the efficacy of ICIs plays an essential role in improving therapeutic outcomes in patients with iCCA. The characteristics of TIME-constitutive cells, which can influence the effectiveness of pharmacotherapy, should be elucidated. However, a detailed TIME analysis is less frequently performed in iCCAs than in other carcinoma types [15,16,17]. Further, the differences in TIME characteristics according to subclassification, which have already been shown to influence surgical treatment strategies [8], have not yet been validated. A better understanding of subclassification-specific TIME may contribute to the development of more effective, individualized treatment strategies and, ultimately, the improvement of iCCA prognosis.

The current study aimed to assess the subclassification-specific TIME characteristics of iCCAs, which can be useful for establishing appropriate pharmacotherapy.

## 2. Materials and Methods

### 2.1. Study Design

This study analyzed 131 patients with iCCA who underwent liver resection, with or without concomitant extrahepatic bile duct resection, at our institution between January 1998 and December 2022. Patients with intraductal papillary neoplasm of the bile duct (IPNB) with invasive carcinoma, classified as intraductal growth-type iCCA, were excluded due to the small number of cases and the fact that this subtype has been reclassified outside of iCCA in the revised WHO classification [1]. In addition, patients with occupational cholangiocarcinoma were excluded, as this entity is characterized by the development of multiple precancerous lesions, leading to simultaneous or metachronous tumors, the clinical and pathological features of which differ significantly from those of typical iCCAs [5,18,19]). Tumors were classified pathologically into small- and large-duct types using the resected specimen. Immunohistochemical analyses were performed to investigate the expression of immune-related molecules in the tumor or tumor-infiltrating cells in each iCCA subclassification.

This study was approved by the Ethics Committee of Osaka Metropolitan University (approval no. 2022-116) and was performed in accordance with the Declaration of Helsinki. All patients provided written informed consent.

### 2.2. Classification of Large- and Small-Duct-Type iCCAs

**Figure 1:**
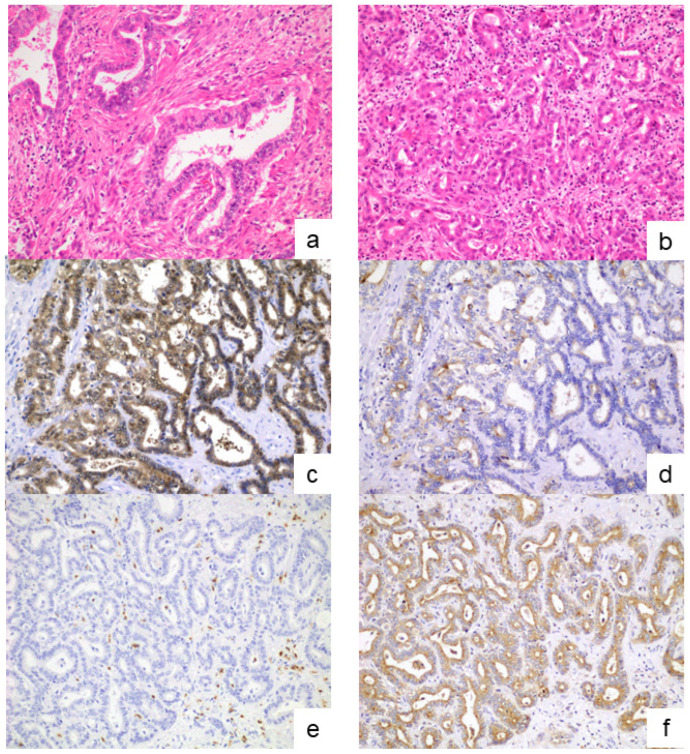
Histopathological findings for small- and large-duct-type iCCAs. Large-duct-type iCCAs showing a large-sized ductal pattern with a long columnar epithelium with mucin production (**a**). Small-duct-type iCCAs comprising small ductal components with low columnar cells without mucin production (**b**). In cases where the iCCA subclassification was challenging to identify based on the findings of H&E staining, S100P immunostaining (**c**,**e**) and SPP1 immunostaining (**d**,**f**) were additionally performed. Predominant positivity to S100P indicated large-duct-type iCCAs (**c**,**d**), and predominant positivity to SPP1 suggested small-duct-type iCCAs (**e**,**f**). (**a**,**b**): H&E staining; (**c**,**e**): S100P immunostaining; and (**d**,**f**): SPP1 immunostaining. Original magnifications: ×200. iCCA, intrahepatic cholangiocarcinoma; H&E, hematoxylin and eosin.

According to the current WHO classification, iCCAs are classified into small- and large-duct types [1]. Formalin-fixed, paraffin-embedded tissue sections stained with hematoxylin and eosin (H&E) were reviewed to determine the subclassification of iCCA. Large-duct-type iCCAs were identified by the presence of large ductal or tubular structures lined by a tall columnar to cuboidal epithelium with mucin production. In contrast, small-duct-type iCCAs consisted of small ductal components (tubular structures with low-columnar-to-cuboidal cells) or ductular components (cuboidal epithelium with ductular or cord-like architecture) lacking mucin production. In 100 out of 131 patients, subclassification was determined based on the H&E staining findings, as well as the main tumor location and gross morphological features (Figure 1a,b).

In cases where the subclassification could not be determined based on the H&E-stained findings (*n* = 31), immunohistochemical analysis was further performed. Primary antibodies against S100P (1:100 dilution, ab133554, Abcam) and SPP1 (1:200 dilution, ab214050, Abcam) were used as the marker characteristics of the large- and small-duct-type iCCAs, respectively [20,21,22]. After deparaffinization, antigen retrieval was performed by microwaving the tissue sections in 10 mmol/L citrate buffer (pH 6.0). Endogenous peroxidase activity was blocked by immersing the sections in 0.3% hydrogen peroxide. Following pretreatment with blocking serum (Dako-Cytomation, Glostrup, Denmark), the sections were incubated overnight at 4 °C with primary antibodies. Subsequently, they were incubated with a secondary antibody conjugated to a peroxidase-labeled polymer using the HISTOFINE system (Nichirei, Tokyo, Japan). Color development was achieved using 3,3′-diaminobenzidine tetrahydrochloride, and the sections were counterstained lightly with hematoxylin. Based on the results of the H&E and immunohistochemical staining, the iCCA cases were classified into small- and large-duct types (Figure 1c–f).

### 2.3. Analyses of the TIME

**Figure 2:**
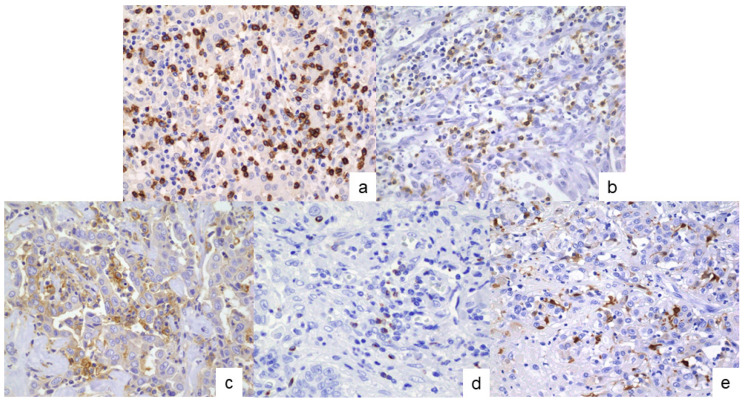
Immunohistochemical assessment of immune-related molecules in the cells of the tumor immune microenvironment. (**a**) CD8, (**b**) PD-1, (**c**) PD-L1, (**d**) CTLA-4, and (**e**) S100 protein. Original magnifications: × 400.

Immunohistochemical analyses targeting CD8, PD-1, CTLA-4, PD-L1, and S100 protein (a dendritic cell [DC] marker) were conducted to investigate the subclassification-specific TIME characteristics. Following deparaffinization, antigen retrieval was performed using an autoclave with a universal HIER antigen-retrieval reagent (Abcam, Tokyo, Japan) for 5 min for PD-L1 staining, and by microwaving in Tris-EDTA buffer (pH 9.0) for 20 min for CD8, PD-1, CTLA-4, and S100 protein staining. After blocking endogenous peroxidase activity, the sections were incubated at room temperature for 1 h with primary antibodies against CD8 (clone C8/144B, 1:20; Dako, Tokyo, Japan), PD-1 (clone NAT105, 1:100; Abcam), CTLA-4 (clone CAL49, 1:200; Abcam), PD-L1 (clone 28-8, 1:500; Abcam), and S100 protein (rabbit polyclonal, prediluted; Nichirei). The sections were then incubated with secondary antibodies using the rabbit-specific IHC polymer detection kit (HRP/DAB, Abcam) for PD-L1 staining, and the Histofine Simple Stain MAX PO (Nichirei) for CD8, PD-1, CTLA-4, and S100 protein staining. Color development was performed using 3,3′-diaminobenzidine tetrahydrochloride, followed by counterstaining with hematoxylin (Figure 2). Negative controls were prepared by replacing the primary antibody with non-immune serum, which resulted in no detectable signal.

The number of CD8-, PD-1-, CTLA-4-, PD-L1-, and S100 protein-positive cells infiltrating the tumor was counted in a high power field. The area within the section with the highest density of positive cells, referred to as the hot spot, was used for the evaluation. Positive cells within the lymph follicles that occasionally formed in the tumor stroma were excluded from the analysis.

The combined positive score (CPS) was evaluated using immunostained sections of PD-L1. Based on a previous study, the CPS was defined as the number of PD-L1-positive cells (including tumor cells, lymphocytes, and macrophages) divided by the total number of viable tumor cells × 100 [23].

### 2.4. Statistical Analysis

The Mann–Whitney U test was used to compare continuous variables between groups, while categorical variables were analyzed using the chi-square test or Fisher’s exact test, as appropriate. Overall survival and recurrence-free survival were estimated using the Kaplan–Meier method and compared using the log-rank test. All statistical analyses were performed using the Statistical Package for the Social Sciences (SPSS; IBM Corp., Armonk, NY, USA).

## 3. Results

### 3.1. Diagnosis of the iCCA Subclassifications

Based on the H&E staining findings, 49 patients were diagnosed with small-duct-type iCCAs and 51 with large-duct-type iCCAs. In the immunohistochemical analysis, 2 of 31 patients presented with diffuse positivity to S100P and negativity to SPP1, which indicated large-duct-type iCCAs. Twenty-four patients showed isolated SPP1 positivity to varying degrees, suggesting small-duct-type iCCA. The remaining five patients exhibited positivity for both S100P and SPP1 at different levels. In these cases, subclassification was determined based on the predominant area of positive staining for S100P and SPP1.

Based on the pathological examination results, 73 patients presented with small-duct-type iCCAs and 58 with large-duct-type iCCAs (*n* = 58).

### 3.2. Differences in the TIME Between Each Subclassification

**Figure 3:**
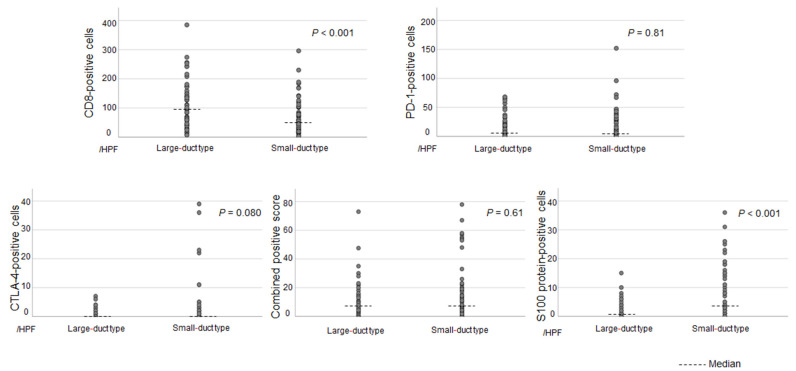
The number of infiltrating cells with immunohistochemically positive expression for each immune-related molecule in large- and small-duct-type iCCA. CD8 was more frequently expressed in large-duct-type iCCAs than in small-duct-type iCCAs. Small-duct-type iCCA had significantly higher expression of S100 protein than large-duct-type iCCA. iCCA, intrahepatic cholangiocarcinoma.

Figure 3 shows the differences in the expression of immune-related molecules in the tumor or tumor-infiltrating cells between each subclassification. The results show no significant differences in the expressions of PD-1 and CTLA-4 and the CPS between the two subclassifications. CD8 was more frequently expressed in large-duct-type iCCAs than in small-duct-type iCCAs (*p* < 0.001). Small-duct-type iCCA had a significantly higher expression of the S100 protein, a marker of DCs, than large-duct-type iCCA (*p* < 0.001). Supplementary Table S1 depicts the median values with ranges for each type.

Patients with a number of S100 protein-positive cells (DC infiltration) > 10 per high-power field were included in the DC-high group. In total, 22 (30%) of the 73 small-duct-type iCCAs were classified as DC-high, and only 1 (1.7%) of the 58 large-duct-type iCCAs were categorized as DC-high. Small-duct-type iCCAs had a significantly higher proportion of DC-high tumors than large-duct-type iCCAs (*p* < 0.001).

### 3.3. Associations Between DC Infiltration and Other Immune-Related Molecules in Patients with Small-Duct-Type iCCAs

**Figure 4:**
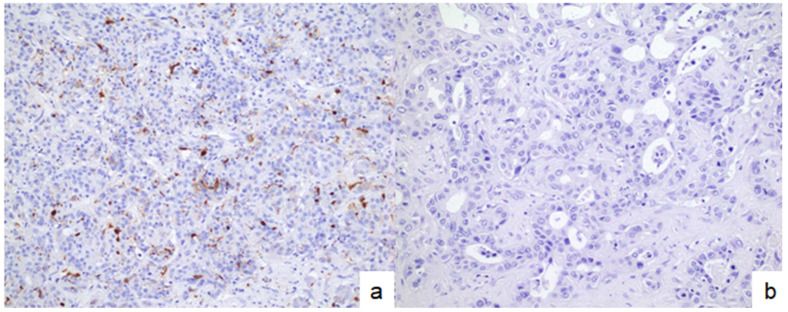
Small-duct-type iCCAs with high and low infiltration of dendritic cells. (**a**) Small-duct-type iCCAs with high infiltration of dendritic cells. (**b**) Small-duct-type iCCAs with low infiltration of dendritic cells. Dendritic cell infiltration was assessed via immunostaining using S100 protein. iCCA, intrahepatic cholangiocarcinoma. Original magnification: × 200.

**Figure 5:**
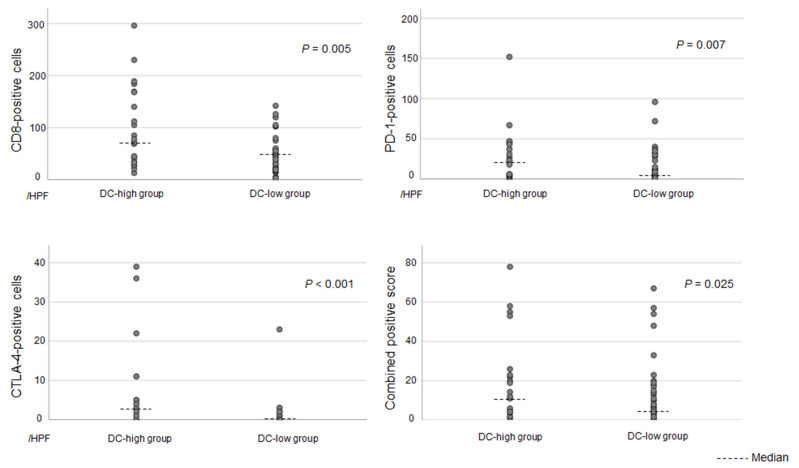
The number of infiltrating cells with immunohistochemically positive expression for each immune-related molecule in the DC-high and DC-low groups in patients with small-duct-type iCCAs. The DC-high group had significantly higher expression of CD8, PD-1, and CTLA-4 than the DC-low group. The DC-high group also had a higher CPS than the DC-low group. DC, dendritic cell; iCCA, intrahepatic cholangiocarcinoma; CPS, combined positive score.

To further investigate the impact of DC infiltration, small-duct-type iCCAs were stratified into DC-high and DC-low groups (Figure 4). Differences in immune-related molecules, including CD8, PD-1, CTLA-4, and PD-L1, between the two groups were evaluated. The DC-high group had significantly higher expression of CD8, PD-1, and CTLA-4 than the DC-low group. Further, the DC-high group had a higher CPS than the DC-low group (Figure 5). Supplementary Table S2 shows the median values with ranges for each group.

### 3.4. Clinicopathological Differences Between DC-High and DC-Low Tumors in Patients with Small-Duct-Type iCCAs

Table 1 presents the clinicopathological characteristics of DC-high and DC-low tumors in patients with small-duct-type iCCAs. The DC-high group had significantly higher frequencies of lymph node metastasis and major hepatectomy than the DC-low group. However, there were no significant differences in other clinical variables between the two groups.

**Figure 6:**
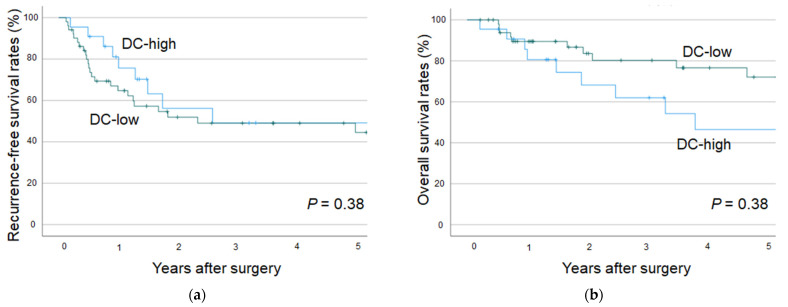
The recurrence-free survival rate (**a**) and overall survival rate (**b**) for small-duct-type iCCAs in the DC-high and DC-low groups. iCCA, intrahepatic cholangiocarcinoma; DC, dendritic cell.

Based on the Kaplan–Meier survival analysis, the recurrence-free survival and overall survival differed between the DC-high and DC-low groups in patients with small-duct-type iCCAs. However, the results did not significantly differ (Figure 6).

## 4. Discussion

Our findings show that the cellular characteristics of the TIME in iCCAs differed according to the subclassification. No significant differences were observed in the expression of PD-1 and CTLA-4 or in the CPS between the small- and large-duct-type iCCAs. However, a subset of small-duct-type iCCAs exhibited high levels of DC infiltration in the TIME. In contrast, such high DC infiltration was rarely observed in large-duct-type iCCAs. Moreover, among patients with small-duct-type iCCAs, the expression of immune-related molecules, including PD-1 and PD-L1, in both tumor cells and tumor-infiltrating immune cells was more frequently observed in the DC-high group than in the DC-low group. These findings emphasize the subclassification-specific differences in the TIME of iCCAs, which may account for the observed variability in the efficacy of pharmacotherapies, including ICIs.

Antitumor immunity begins with the recognition of tumor antigens by antigen-presenting cells, including DCs, which leads to the induction and activation of immunocompetent cells such as CD8-positive T cells. In response to this induced antitumor immunity, tumors evade the host immune response by expressing immune-exhausting molecules, such as PD-1, PD-L1, and CTLA-4, on the surface of tumor cells or tumor-infiltrating cells in the TIME, and they grow and develop [24,25,26,27,28]. Tumor antigen release is caused by various mechanisms, and the tumor mutation burden (TMB) has one of the most representative roles [29,30]. A high TMB suggests that the tumor cells carry numerous genetic mutations. If the TMB is high, neoantigens are produced more frequently by the tumor, which are easily recognized by the immune system. Thus, they induce the expression of immune-related molecules to evade the anti-tumor immunity [29,30]. iCCAs are generally associated with tumors with a low TMB, which often results in a low expression of immune-related molecules in the TIME (immune-cold tumor) and can influence the efficacy of ICI therapy [31]. The current study found that the degree of infiltration by DCs, a key antigen-presenting cell, varied significantly between the two subclassifications. The expression of other immune-related molecules in the TIME was significantly more common in small-duct-type iCCAs with high DC infiltration than in those with low DC infiltration. Based on these findings, in some small-duct-type iCCAs, a specific mechanism may upregulate immune-related molecules in the TIME to evade tumor immunity activated by DC-mediated antigen presentation. Further investigation into the mechanisms that trigger tumor antigen release leading to DC infiltration, including the potential role of the TMB, should be conducted.

In recent years, the potential therapeutic benefit of ICIs has been observed across various cancers [9,10,11,12,13]. As mentioned previously in this manuscript, almost all iCCAs are characterized as immune-cold tumors [31]. Thus, the efficacy of single-agent ICIs remains limited. In pharmacotherapy using ICIs for iCCAs, combination therapy with cytotoxic chemotherapy may be recommended as it can enhance tumor necrosis, tumor antigen release, and associated immune editing, which can be regulated by ICIs, leading to improved therapeutic outcomes [12]. Based on this rationale, the addition of ICIs to gemcitabine and cisplatin had promising outcomes compared with the control in the TOPAZ-1 and KEYNOTE-966 trials [12,13]. Nonetheless, previous studies have reported that systemic chemotherapy, including anti-PD-1 antibody therapy, has a lower efficacy against large-duct-type iCCAs [14]. In addition, patients with KRAS gene mutations, which are more common in large-duct-type iCCA [32,33,34,35], are less responsive to pharmacotherapy [14]. The association between the efficacy of ICIs and the expression of immune-related molecules in the TIME remains unclear [14,36,37,38,39]. Nevertheless, the differences in pharmacotherapeutic efficacy among iCCA subclassifications may be associated with the subclassification-specific differences in the TIME, as shown in the current study. Taken together with previous findings [14], our results show that the subclassification of iCCA might play an essential role in determining appropriate pharmacotherapy. Further, small-duct-type iCCAs might include a subset of tumors with greater responsiveness to pharmacotherapy, including ICI therapy.

In the current study, there were no significant differences in the long-term prognosis or background factors between the DC-high and DC-low groups in patients with small-duct-type iCCAs. This cohort consisted of a limited number of cases and included only resectable iCCA. Therefore, various biases, including the treatment era, could not be fully excluded. Accordingly, definitive conclusions regarding detailed prognostic differences between the two groups cannot be drawn from the present findings. However, interestingly, although small-duct-type iCCAs are associated with a lower risk of lymph node metastasis compared with large-duct-type ICCs [8], the DC-high group exhibited a higher frequency of lymph node metastasis than the DC-low group. Furthermore, although no significant differences were observed between the two groups in terms of tumor size, tumor number, or pathological vascular invasion, the frequency of major hepatectomy was significantly higher in the DC-high group. These results should be reinvestigated via large-scale studies, because the current study had a small sample size. Nevertheless, these results may be attributed to aggressive tumor malignancy induced by cancer immune editing triggered by DC infiltration. The variable characteristics of the TIME may be of potential utility in choosing the appropriate treatment strategy, including surgery. Lymph node metastasis is a particularly important factor influencing poor prognosis in iCCA [40,41]. If ICIs are effective in such groups, they may significantly improve patient prognosis, particularly in small-duct-type iCCAs.

This study has several limitations. First, it involved conducting a retrospective analysis at a single institution. This might have introduced selection bias and limited the generalizability of the findings. However, despite the relatively small sample size, significant differences in the TIME, particularly in DC infiltration, were observed between the iCCA subclassifications. These findings may provide important insights into the heterogeneity of the TIME in iCCA. Second, differences in TIME characteristics were observed between each ICC subclassification. Nevertheless, the current study could not assess the impact of these variations on the efficacy of ICIs. If the association between the expression of immune-related molecules and the effect of ICIs is confirmed, the assessment of DC infiltration using the antibody for the S100 protein could be a possible predictive biomarker for the efficacy of ICIs. Therefore, future studies with larger cohorts and clinical trials incorporating ICI therapy must be performed to validate our findings and further explore the role of TIME heterogeneity in iCCA treatment strategies. Finally, subclassification-specific TIME characteristics were identified. Nonetheless, the cause of these discrepancies is currently unknown, and the background characteristics did not present with significant distinctions. Therefore, further studies should be conducted to determine the causes of differences in the TIME via genetic analysis of each group.

## 5. Conclusions

The current study identified subclassification-specific TIMEs in ICCs. Strong DC infiltration was observed in a subset of small-duct-type iCCAs. This may reflect an immunosuppressive TIME that enables evasion of antitumor immune responses initiated by DC-mediated antigen presentation. The diversity in the background factors and anatomical origins of iCCAs not only contributes to the formation of distinct subclassifications with different clinicopathological characteristics, but also influences the composition of the TIME. This result may contribute to establishing appropriate pharmacotherapy for each subclassification.

## Figures and Tables

**Table 1:** Clinicopathological differences between the DC-high and DC-low groups in patients with small duct-type iCCAs.

Variables	DC-High Group (*n* = 22)	DC-Low Group (*n* = 51)	*p*-Value
Age, years	70 (55–89)	68 (32–82)	0.38
Sex, male/female	18/4	37/14	0.56
Laboratory data results			
T-Bil level, mg/dL	0.6 (0.3–1.6)	0.6 (0.1–22.7)	0.43
Albumin level, g/dL	4.0 (3.3–4.5)	4.2 (2.7–4.8)	0.35
AST level, U/L	33 (17–164)	29 (11–164)	0.60
ALT level, U/L	24 (9–208)	27 (7–208)	0.76
CEA level, ng/mL	3.4 (1.1–9.2)	3.4 (0.7–56.9)	0.88
CA19-9 level, U/mL	16 (5–1554)	28 (2–1392)	0.82
Chronic liver disease	13	27	0.75
Viral hepatitis	5	15	0.58
Positive for HBsAg	1	4	0.99
Positive for HCVAb	4	11	0.99
Metabolic-associated steatohepatitis	5	6	0.23
Alcoholic hepatitis	3	8	0.99
Development of synchronous or metachronous HCC	1	9	0.26
Preoperative chemotherapy	2	3	0.63
Tumor diameter, cm	3.4 (1.2–7.3)	3.8 (0.4–12.5)	0.50
Macroscopic classification, MF/PI	21/1	51/0	0.30
Multiple lesions, n	3	14	0.15
Perihilar invasion	10	12	0.17
Adjuvant chemotherapy	9	19	0.77
Major hepatectomy	15	12	0.043
Pathological findings			
Liver cirrhosis	1	6	0.67
Perineural invasion	3	5	0.69
Microvascular invasion	10	18	0.41
Lymph node metastasis	7	6	0.040
Tumor invasion at the surgical margin	2	5	0.99

Median (range). DC, dendritic cell; iCCA, intrahepatic cholangiocarcinoma; T-Bil, total bilirubin; AST, aspartate aminotransferase; ALT, alanine aminotransferase; CEA, carcinoembryonic antigen; CA19-9, carbohydrate antigen; HBsAg, hepatitis B surface antigen; HCVAb, hepatitis C virus antibody; HCC, hepatocellular carcinoma; MF, mass-forming; PI, periductal infiltration.

## Data Availability

All data generated or analyzed during this study are included in this article and its Supplementary Material files. Further enquiries can be directed to the corresponding author.

## Supplementary Materials

The following supporting information can be downloaded at: https://www.mdpi.com/article/10.3390/cancers17132082/s1, Table S1: The number of positive cells for each immune-related molecule in large- and small-duct-type iCCA; Table S2: The number of positive cells for each immune-related molecule in the DC-high and DC-low groups in patients with small-duct-type iCCAs.

## Abbreviations

The following abbreviations are used in this manuscript:

DC	Dendritic cell
H&E	Hematoxylin and eosin
iCCA	Intrahepatic cholangiocarcinoma
ICI	Immune checkpoint inhibitor
TIME	Tumor immune microenvironment
TMB	Tumor mutation burden
