# Supplementary material for: Subclassification-Specific Tumor Immune Microenvironment in Intrahepatic Cholangiocarcinoma: Implications for Appropriate Pharmacotherapy
Source: Cancers (Basel). 2025 Jun 21;17(13):2082. doi: 10.3390/cancers17132082 (PMC12248564; doi:10.3390/cancers17132082)
Supplement: Supplementary file 1 [file cancers-17-02082-s001.zip › Supplementary Table S1.pdf]

Supplementary Table S1. Number of positive cells for each immune-related molecules between large- and small-duct-type ICCs.

	Total	Large-duct-type ICCs n = 58	Small-duct-type ICCs n = 73	*p*-value
CD8-positive cells, n	58 (3-385)	100 (7-385)	46 (3-296)	< 0.001
PD1-positive cells, n	6 (0-152)	6 (0-68)	6 (0-152)	0.811
CTLA4-positive cells, n	0 (0-39)	0 (0-7)	0 (0-39)	0.080
Combined positive score	6 (0-78)	6 (0-73)	6 (0-78)	0.605
S100-positive cells (DC infiltration), n	2 (0-36)	1 (0-15)	3 (0-36)	< 0.001
DC-high (HPF > 10), n	23	1	22	< 0.001

Median (range)

ICC, intrahepatic cholangiocarcinoma; DC, dendritic cell; HPF, high power field.
