# Supplementary material for: Subclassification-Specific Tumor Immune Microenvironment in Intrahepatic Cholangiocarcinoma: Implications for Appropriate Pharmacotherapy
Source: Cancers (Basel). 2025 Jun 21;17(13):2082. doi: 10.3390/cancers17132082 (PMC12248564; doi:10.3390/cancers17132082)
Supplement: Supplementary file 1 [file cancers-17-02082-s001.zip › Supplementary Table S2.pdf]

Supplementary Table S2. Number of positive cells for each immune-related molecules between the DC-high and DC-low groups in patients with small-duct-type ICCs.

	**Total**	**Small-duct-type DC-high group (n = 22)**	**Small-duct-type DC-low group (n = 51)**	***p* value**
CD8-positive cells, n	46 (3-296)	75 (13-296)	43 (3-142)	0.005
PD1-positive cells, n	6 (0-152)	21 (0-152)	5 (0-96)	0.007
CTLA4-positive cells, n	0 (0-39)	3 (0-39)	0 (0-23)	< 0.001
Combined positive score	6 (0-78)	12 (1-78)	6 (0-67)	0.025

Median (range)

ICC, intrahepatic cholangiocarcinoma; DC, dendritic cell; HPF, high power field.
